# Cardioneuroablation: the known and the unknown

**DOI:** 10.3389/fcvm.2024.1412195

**Published:** 2024-07-26

**Authors:** A. Marrese, R. Persico, E. Parlato, D. Faccenda, A. Salucci, G. Comparone, V. Pergola, G. Ammirati, L. Addeo, C. Fonderico, L. Cocchiara, A. Volpe, P. Visconti, A. Rapacciuolo, T. Strisciuglio

**Affiliations:** Department of Cardiology, University of Naples Federico II, Naples, Italy

**Keywords:** cardioneuroablation (CNA), vasovagal syncope, atrioventricular block, high-frequency stimulation (HFS), fractionated electrograms, cardioinhibitory syncope

## Abstract

Cardioneuroablation (CNA) is a novel interventional procedure for the treatment of recurrent vasovagal syncope (VVS) and advanced atrioventricular block secondary to hyperactivation of vagal tone in young patients. By damaging the cardiac parasympathetic ganglia, CNA seems to be able to mitigate and/or abolish the excessive vagal activity and improve patients’ outcome. This review is intended to give a detailed and comprehensive overview of the current evidences regarding (1) the clinical applications of CNA (2) the identification of ablation targets and procedural endpoints (3) the medium-long term effect of the procedure and its future perspectives. However, clinical data are still limited, and expert consensus or recommendations in the guidelines regarding this technique are still lacking.

## Introduction

Vasovagal syncope (VVS) is the prevailing cause of syncope and it is due to an imbalance between the sympathetic and parasympathetic components of the autonomous nervous system (ANS). An excessive parasympathetic response to a trigger may provoke a syncope as the result of bradycardia and hypotension leading to cerebral hypoperfusion. According to the VASIS classification, VVS is generally divided into 3 types, based on the underlying mechanism: mixed (I), cardioinhibitory (IIa-b), and vasodepressive (III) (1).

These syncopal episodes are generally benign and usually occur in healthy persons, but if recurrent they can significantly worsen the patients’ quality of life, especially if unresponsive to conservative measures, such as patient education to execution of hand-gripping and leg crossing maneuvers at the onset of symptoms.

According to the latest guidelines, in case of recurrent cardioinhibitory syncope pacing is indicated only in patients aged >40 years, thus CNA could be a favorable therapeutic option in younger patients (1, 2).

**Figure 1:**
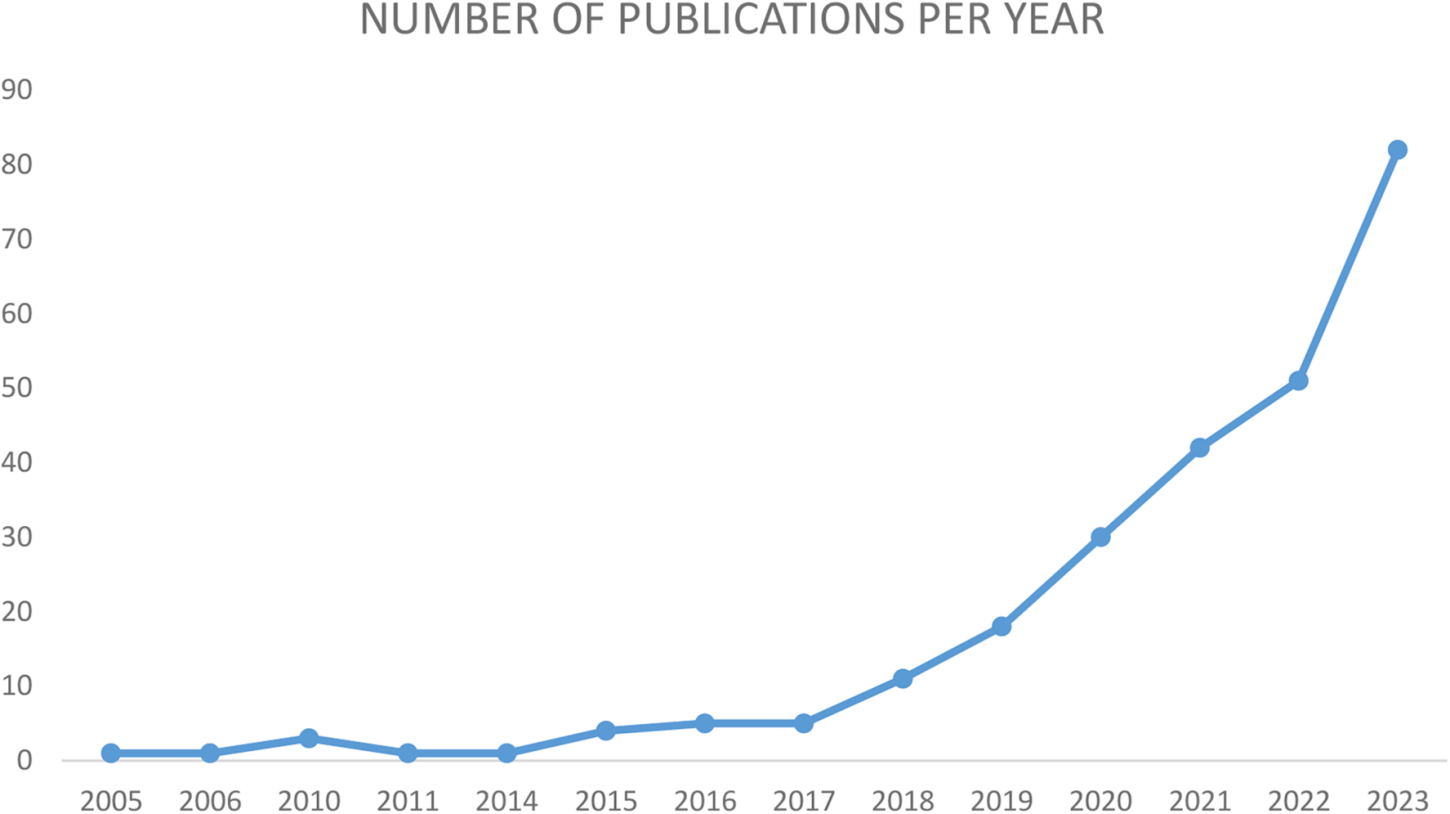
Increasing number of publications per year regarding CNA.

Cardioneuroablation (CNA), consisting of vagal denervation in correspondence of AF nest at the pulmonary veins antral regions, was originally evaluated as a potential integration to atrial fibrillation (AF) ablation (3). Pachon et al. were the first to propose the CNA as a therapeutic strategy for the management of syncope or bradyarrhythmias associated with the hyperactivity of the vagal tone (4), by ablating the cardiac ganglionated plexuses and mitigating the underlying parasympathetic overdrive (5). Furthermore, some evidences suggest a role for CNA also for the treatment of functional atrio-ventricular block (AVB) and sinus node dysfunction (SND) (6, 7). As recent studies show convincing results, the number of electrophysiologists performing this type of procedure and thus the number of patients undergoing this treatment are increasing and in parallel also the number of publication on this topic has seen an exponential growth in the last decade (Figure 1).

However, despite the great interest for this novel technique, there aren't at the moment recommendations in guidelines nor Expert Consensus about CNA. Remarkably, there is still high variability either in patients’ selection as well as in procedures performance and outcomes evaluation. There is moreover a lack of large RCTs demonstrating the superiority of this kind of therapy on the conventional pharmacological and non pharmacological therapy.

This review aims at elucidating (1) the rationale and the clinical applications of CNA (2) the identification of ablation targets and procedural endpoints (3) the medium-long term effect of the procedure and its future perspectives.

### Anatomy and functioning of the autonomic nervous system

The autonomic cardiac nervous system is composed by the central nuclei in the brainstem, and 2 peripheral main cells, the preganglionic and the postganglionic neurons. Furthermore, the efferent ANS is divided in two components, parasympathetic and sympathetic, having opposite functions and a different structural organization. While the sympathetic postganglionic neurons with their long axonal endings are mainly located in the paravertebral sympathetic chain, on the other hand the parasympathetic postganglionic neurons with very short axonal endings are organized in ganglionated plexuses (GPs), that are situated in the atrial wall. Therefore, the parasympathetic ganglia can be more easily destroyed from the endocardium with radiofrequency ablation, and when the re-innervation occurs, this is a slow process as it requires the differentiation of interneurons. Some sympathetic fibers are also localized in the fat pad surrounding the heart and thus these can be damaged during ablation, however the sympathetic re-innervation is easier as it derives from the regeneration of the same neurons (8–10).

Noteworthy, myocardial activity is not dependent on the autonomic innervation: thus, where the skeletal muscle atrophies after denervation, the cardiac muscle preserves its activity and function regardless of the denervation.

The anatomical localization of GPs has been studied and described by different Authors.

**Figure 2:**
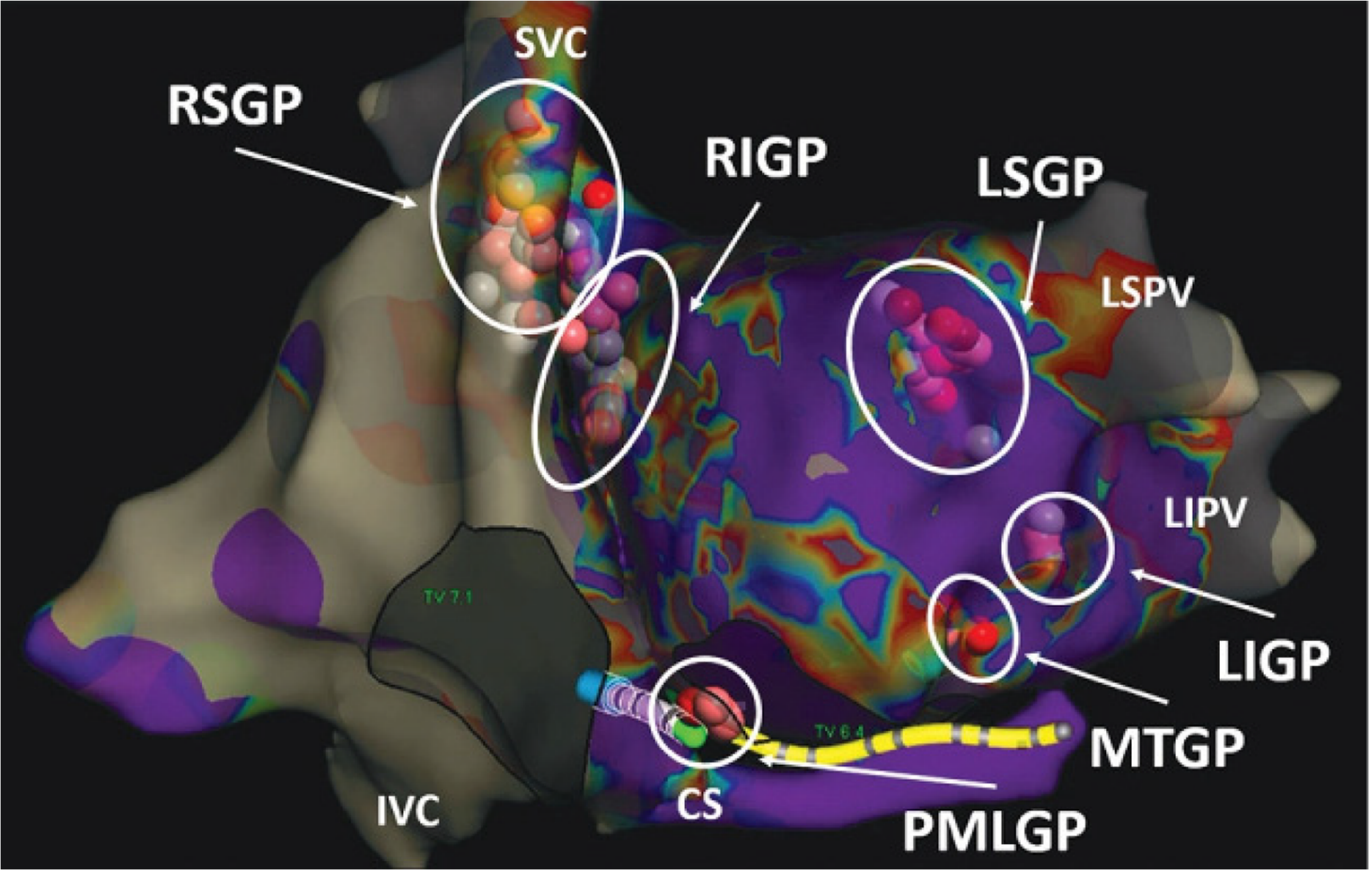
Localization of right and left ganglia. Courtesy of Aksu et al. (doi: 10.15420/aer.2022.37).

Cardiac parasympathetic ganglia are usually localized as follows (Figure 2, courtesy of Aksu T. et al). The right superior ganglionated plexus (RSGP) is in between the superior vena cava and the right superior pulmonary vein. From this ganglion, most of the efferent parasympathetic fibers travel into the atria through the medial part of superior vena cava and the aortic root (11). The right inferior ganglionated plexus (RIGP) is positioned between the right pulmonary veins and the right atrium (12). The posteromedial left ganglionated plexus (PMLGP) is located in the posterior portion of the interatrial septum, between the wall of the left atrium, the inferior vena cava, and coronary sinus ostium, even if a part of the fibers related to this GP may extend to the left atrial side. These three ganglia are sometimes named as ganglion A, B and C respectively (13). The left superior ganglionated plexus (LSGP) is situated between the left atrial appendage and the left superior pulmonary vein. The left inferior ganglionated plexus (LIGP) is located inside the fat pad anterior to the left inferior pulmonary vein (14, 15). Lastly, the Marshall ligament is currently considered as part of the cardiac ANS as contains cholinergic vagal fibers and is named Marshall tract ganglionated plexus (MTGP) (16).

However, the specific GPs locations vary significantly from individual to individual, thus their described anatomical locations are not always accurate, although the localization of the right ganglia is quite constant (17).

Several studies indicate that the sinus atrial node (SAN) and the atrio-ventricular node (AVN) are respectively innervated by the RSGP, mainly composed by the right vagus nerve fibers, and by the RIGP/PMLGP, as the final pathway of left vagus nerve to AVN, thus allowing a selective destruction of these ganglia in case of bradyarrhythmias related to atrio-ventricular block or sinus node dysfunction, and thus sparing the remaining autonomous system (18–22).

### Clinical applications of the CNA: patients selection

Even if there are not universally accepted criteria for patients’ selection, CNA could be proposed in case of recurrent syncopal episodes per year and failure of conservative strategies, for patients in whom, due to age <40 years, pacing would not be desirable. Anyway the definition of the underlying mechanisms of syncope is essential: in fact, in case of vasodepressive syncope, CNA is unable to effectively prevent the recurrence of syncopal episodes, whereas CNA by avoiding excessive vagal influence on sinoatrial node or atrio-ventricular node (23), may counteract the cardioinhibitory syncope and partially also the mixed forms.

The head-up tilt test (HUTT) is the most useful test to identify patients with an autonomic substrate for VVS. According to the VASIS classification, the HUTT response are the following: type 1, mixed response, for a reduction in both blood pressure and heart rate (>40 bpm or <40 bpm but for <10 s); type 2A, cardioinhibitory for heart rate reduction <40 bpm for >10 s, but without asystole; type 2B, cardioinhibitory with asystole >3 s; and type 3 (vasodepressor response) (24). Patients affected by cardioinhibitory syncope, especially those with asystole, but also those with type 2A, may be good candidates to CNA. While there is still no clear indication of CNA for type 3, due to its vasodepressive nature, the CNA may be beneficial in some patients with type 1 (19, 25, 26).

The HUTT is a widely adopted diagnostic test to induce syncope, however the results are poorly reproducible and this may significantly limit its value. Furthermore, even when the HUTT result suggests a vasodepressive nature of the syncopal episode, in some patients also implanted with a loop recorder (ILR), a cardioinhibitory episode can be recorded, leading to a change in therapeutic approach. Thus, an accurate selection of patients undergoing to CNA is crucial (27, 28). The demonstration of asystole during a spontaneous syncope, documented by ILR, and the exclusion of susceptibility to hypotension via 24-hours blood pressure Holter monitoring may help to identify those patients who may benefit the most from a CNA.

Atropine test is frequently used for the selection of patient candidate to CNA. Atropine blocks cardiac muscarinic receptors and thus vagal activity, therefore during the infusion of this agent normally there is an increase in HR. Particularly, an acceleration of HR over 90 bpm or an increase over 25% than baseline after intravenous atropine bolus (Dose: 0,04 mg/Kg, Maximum dose 3 mg) suggest a prevalence of the parasympathetic system (25, 29). In case of a negative response to atropine test, the CNA should be discouraged and the implant of a pacemaker should be preferred, as it indicates a poor effect of vagal tone on heart rhythm (30).

Furthermore, heart rate variability (HRV) and deceleration capacity (DC) are two non-invasive parameters that can be derived by the ECG monitoring through specific algorithms. The first, derives from the RR interval variability on ECG, which is often reduced in patients with VVS story; the second, derives from HRV analysis, and values >7.5 ms, suggest an increased parasympathetic overactivity (28–30).

### Procedural aspects of the CNA

#### Epicardial ganglia localization during the ablation procedure

Different methods have been used for ganglionic plexuses localization: fractionated EGM evaluation, high frequencies stimulation (HFS), anatomic approach and recently the computed-tomography and the Iodine 123 metaiodobenzylguanidine (MIBG) SPECT.

**Figure 3:**
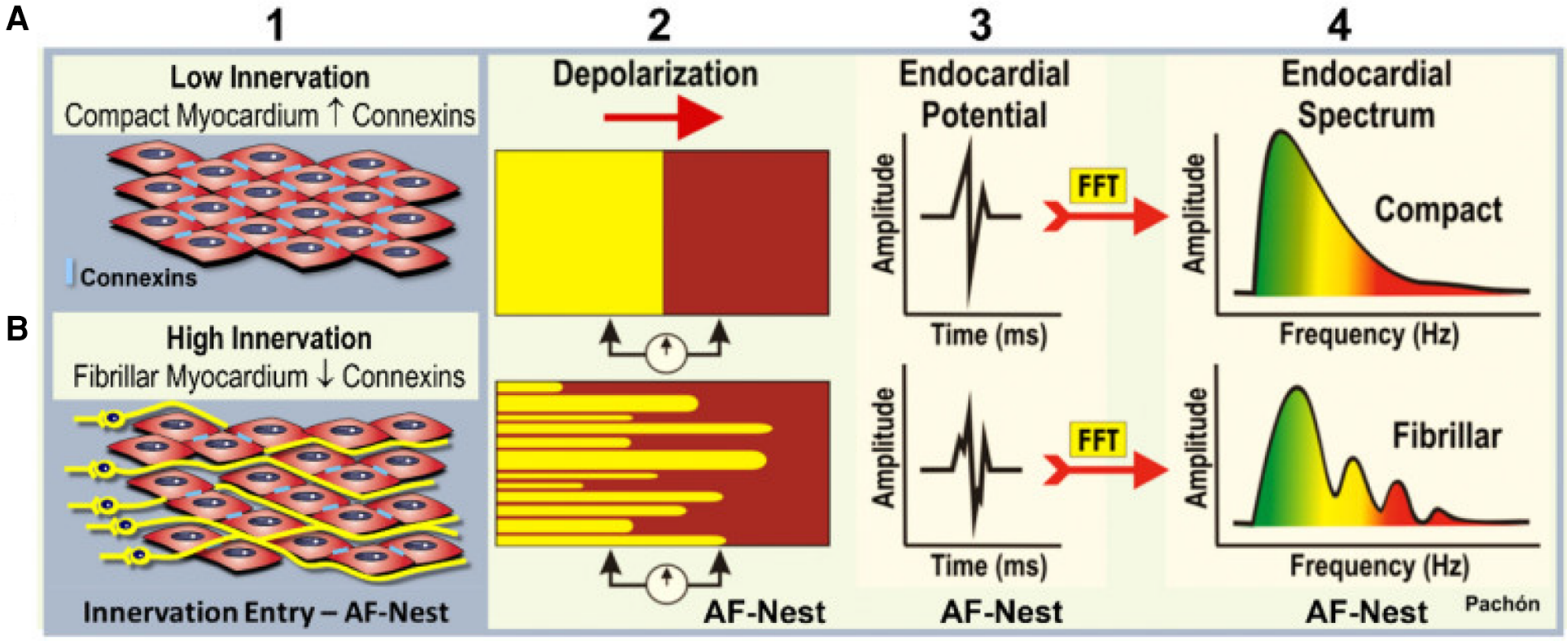
Mapping of endocardial potentials to find ganglia through the fast Fourier transformation. (**A**) Compact myocardium has a poor innervation, and the electrical conduction is homogeneous and paracellular due to the presence of connexins. That result in a clear and delineated endocardial potential. (**B**) Fibrillar myocardium: at the level of the neuro-myocardial interface, as in correspondence of the cardiac ganglia, the neural fibers determine a spectrum of highly fragmented endocardial potentials. *Courtesy by Pachon et al.* (32).

In 2004 Pachon et al. proposed a new method for endocardial potentials study during AF ablation, applying the Fast-Fourier transformation (FFT), a mathematical algorithm that enables the visualization of any wave as the sum of several sinus waves which create a frequency spectrum. In this way, they found that the myocardial organized conduction has a narrow frequency spectrum of signal, and described two different types of atrial myocardium (Figure 3): the compact one, presenting a homogeneous range with one dominant frequency around 40 Hz and with uniform conduction, and the fibrillar one, presenting a heterogeneous range with several fraction having frequencies >100 Hz (31, 32). They found that in the absence of cardiopathy, fibrillar spectrum can be localized not only in correspondence of fibers of cardiac conduction system, but also in many other localizations, mostly near cardiac para-ganglia (33).

Therefore, through a specific software that automatically identifies these fractionated electrograms (EGM) and tag them (Ensite Precision system©), it is possible to localize the parasympathetic ganglia during electroanatomic mapping of the left (LA) and right atrium (RA) (34, 35). The AF nests are atrial sites with high frequencies components, where it is possible to localize ganglia sites by setting the filters between 200 Hz and 500 Hz, respectively, and by recognizing the fragmented EGM with ≥4 deflections (36).

During routine atrial mapping, usually performed through a decapolar catheter for right atrium and a steerable quadripolar catheter for left atrium, EGMs are classified into 3 groups according to their amplitude and number of deflections: (1) normal atrial EGM, with a deflection number <4; (2) low-amplitude fractionated EGM (LAFE), with ≥4 deflections and amplitude <0.7 mV; and (3) high-amplitude fractionated EGM (HAFE), with ≥4 deflections and an amplitude ≥0.7 mV (37). The presence of HAFE or LAFE pattern, at a sweep speed of 400 mm/s, recorded in a region near to probable localization of GPs are tagged as ablation targets.

CNA may be performed solely by anatomic landmarks, delivering radiofrequency energy (RF) in presumed locations of atrial epicardial fat pad, however this approach can easily be inaccurate, especially for the left GPs, due to interpatient variability. On the other hand, more recently enhanced anatomical approaches have been proposed and these include a CT-based identification of ganglia to improve the precision of RF delivery during CNA (38, 39), and the use of D- SPECT [123I]I-metaiodobenzylguanidine cardiac SPECT to provide anatomical quantification of autonomic nervous system structures (40).

Also high frequency stimulation (HFS) has been described for parasympathetic ganglia localization. Usually, HFS is delivered to GPs sites with these parameters: voltage of 10–20 V, amplitude of 30 mA, frequency of 20 Hz, and pulse duration of 5s (41). This stimulation may determine two types of atrial response: a prolongation of the PR or RR intervals by 50%, a transient ventricular asystole, or an AV block, which is defined as vagal response (VR), or the absence of changes in the PR or RR intervals, which is defined as a normal response (42, 43). The first response is typical of the GP sites whereas the second one reflects a normal atrial myocardium. The major limitations of HFS are the following: atrial fibrillation induction, need of general anesthesia, and inadvertent excitation of atrial wall nociceptors that leads to negative dromotropic or chronotropic effects. Additionally, because a significant portion of autonomic nerve fibers are located distant from the major GPs, the stimulation of these fibers, such those close to the pulmonary vein antrum, may lead to a false positive response.

Beyond the described methods, the best marker of the accurate location of the GPs is the immediate response to RF application, with asystole or a significant increase of the RR intervals occurring within a few seconds of application.

#### How to perform the ablation

The procedure is generally performed under general anesthesia to guarantee a high vagal tone during the procedure and to increase patients’ tolerance, however the use of halogenated gas should be preferred, as with these drugs there is no risk of blunting the response to CNA. The use of conscious or deep sedation (midazolam and midazolam + propofol, respectively) is associated with a reduction of vagal response during ablation of right and left GPs (44).

The transseptal puncture is necessary in case the ablation is performed also in the left atrium.

The mapping is generally performed using 3D navigation systems. Once the 3D atrial map is reconstructed, the radiofrequency energy is delivered with irrigated catheters with a power commonly limited to 40 W (45) in cycle of 30 s or more. The ablation is commonly performed until the HAFE pattern is significantly reduced (a peak to peak distance <0.05 mV in bipolar electrogram) and vagal response to HFS disappears (32, 46–48).

### Right or left atrium: where to start?

Chiou et al. demonstrated that several vagal fibers directed to the atrionodal sinus and the atrioventricular node travel across a fat pad close to the RSGP, in close proximity with superior vena cava (SVC), right pulmonary artery and aortic root (Ao). This so called “SVC-Ao fat pad” could be considered as a connection zone of all vagal fibers (11).

The ablation of the RSGP first was associated to significant modifications of vagal responses during the procedure, in term of changes in HR and blood pressure (49, 19).

Therefore, ablating the RSGP first, makes difficult the evaluation of subsequent ganglia denervation (37, 44). Aksu et al. therefore recommend a precise sequence of ablation, which implies the right-sided ganglia last: (1) LSGP, (2) LIGP and MTGP, (3) RSGP, (4) and RIGP (30). Anyway, there is still not a clearly approved order for ganglia denervation.

Brignole et al. proposed the execution of CNA via the right atrial approach alone, through the ablation of the sole RSGP in patients with recurrent cardioinhibitory syncope, and the ablation of the LIGP in case of functional AVB, exclusively or in addition to RSGP, considering these ganglia as the anatomical common pathway of cardiac parasympathetic fibers (50). Both ganglia are in the close proximity of the interatrial septum, and may be reached by ablation from right atria, avoiding a biatrial approach, which needs the transseptal puncture, thus reducing the duration of the procedure and the related risks, as embolization or pericardial effusion (12, 51, 52). Nevertheless, the right atrial ablation may be effective for the denervation of sinus node GPs, but may be not enough for the AV node GPs. Even if the ablation solely from the right atrium may be effective for cardiac nerve modulation and to prevent syncope recurrences, the ablation from both atria seems able to reach a more extensive denervation when it is quantitatively evaluated by extracardiac vagal stimulations (53–55).

Furthermore, the recent ROMAN2 study compared the acute effectiveness of the ablation from right vs. left atrium: when the ablation was performed in the right atrium, it was associated to a lower rate of complete vagal denervation, needing the cross-over to the other atrium to achieve an effective denervation (65% vs. 20%) (56).

However, there are yet no studies of head-to-head comparison of long-term results between right-sided, left-sided and biatrial approach for CNA, necessary to confirm the reproducibility of the right-sided alone ablation (48).

### Ablation endpoint

The CNA appears to be more effective when associated with an extensive vagal denervation, as the probability of re-innervation is lower, however there is still not a clear strategy to evaluate that, thus it would be more appropriate to name it cardioneuromodulation rather than cardioneuroablation. Furthermore, a complete vagal denervation is also not desirable for the potential harmful effects, as discussed later in the text.

The HFS, repeated at each ablation site, is used to assess the elimination of a positive VR. The latter may be considered a suitable ablation endpoint, indeed further ablation is needed when VR remains positive. However, HFS is neither sensitive nor specific and, cannot predict the long-term effect of the ablation.

The absence of HR changes during atropine infusion is also considered a proof of satisfying vagal denervation. However, atropine should be given only at the end of the procedure, due to its long-lasting effect. Pachon et al, proposed a novel approach to evaluate the efficacy of vagal denervation during CNA, by performing an extracardiac vagal stimulation (ECVS), during general anesthesia, via the internal jugular vein, taking advantage of the great proximity of the vagus to that vein (34). By advancing a quadripolar irrigated ablation catheter to jugular foramens bilaterally, the stimulation with an amplitude of 1 V/kg, maximum 70 V, with a width of 50 ms, and a frequency of 50 Hz for 5 s may elicit the VR (33). When the response is still positive after ablation this suggests an incomplete vagal denervation. The ECVS can be performed during CNA or at the end of the procedure. Ultrasound can be used to easily localize the vagus nerve, which appears thin and hyporeflective along the venous wall: some evidences showed that ultrasound-guided ECVS more frequently than fluoroscopy-guided ECVS enables to reach vagal positive response (57, 58).

It has recently been confirmed the superiority of an ECVS-guided approach in comparison to a CNA relying on anatomical landmarks and fractionated potentials mapping: indeed, performing ECVS before ablation to evaluate basal responses, during the procedure and after every cycle of radiofrequency to confirm the success of denervation, was associated to a lower rate of syncope recurrence compared to CNA performed without ECVS guidance (59).

Anyway, ECVS may have different effects depending from which side the stimulation is performed: usually, the right vagus nerve is more commonly stimulated because of the easiness of access of right jugular vein, but the AVN is mainly controlled by the left vagus nerve, thus the absence of VR to right ECVS doesn't guarantee always an effective denervation (60). Thus, bilateral vagal nerve stimulation should be performed to ensure the clinical efficacy of CNA, especially in patients with a relevant AVN disfunction.

Therefore, ECVS is an interesting method to evaluate vagal denervation, but it lacks of a long-term follow-up in order to make any comparison with the other methods.

### Ablation outcomes and safety

CNA appears to be able to significantly reduce the recurrence of cardioinhibitory syncope (Table 1). A recent meta-analysis proves a freedom from syncope recurrence of 92% (57). A RCT published by Piotrowski et al. in 2022 compared syncope recurrence after CNA vs. non-pharmacological optimal therapy in 48 patients: they reported 8% recurrence in CNA patient group vs. 54% recurrence in controls group, during a 2-years follow-up (69). In a case report, the recurrence of syncope after CNA was associated to a vasodepressor response at HUTT, thus confirming the success of cardiac denervation, although the persistence of symptoms (70).

**Table 1:** Freedom from syncope recurrence after CNA, as reported by the current literature.

Authors	Year of publication	Number of patients	Type of approach	Follow-up (Months)	% of Success at follow-up	Approach for Ganglia localitation
Pachon et al. (32)	2005	21	Both atria	9.2 ± 4.1	100%	Spectral mapping
Pachon et al. (25)	2011	43	Both atria	45.1 ± 22	93%	Spectral mapping
Yao et al. (61)	2012	10	Left atria	30 ± 16	100%	HFS
Sun et al. (62)	2016	57	Left atria	36.4 ± 22.2	91%	HFS/Anatomic
Rivarola et al. (63)	2017	14	Both atria	22.5 ± 11.3	71.4%	EAM
Aksu et al. (37)	2019	20	Both atria	12	90%	EAM/HFS + Spectral analysis
Hu et al. (19)	2019	115	Left atria	21.4 ± 13.1	92%	HFS and or Anatomic
Calo et al. (48)	2020	18	Right atria	34.1 ± 6.1	83%	Anatomic
Aksu et al. (64)	2020	51	Both atria	11	94%	HFS + Spectral analysis
Pachon et al. (65)	2020	83	Both atria	40	80%	Anatomic + FEGM
Huang et al. (66)	2020	49	Left atria	17.8 ± 10.5	92%	EAM
Debruyne et al. (26)	2021	51	Right atria	12	95%	CT-guided
Piotrowski et al. (67)	2021	20	Both atria	12	100%	Anatomic + FEGM
Joza et al. (68)	2024	6	Both atria	13.4	67%	FEGM + HFS

HFS, high-frequency stimulation; EAM, electroanatomic mapping; FEGM, fractionated electrograms.

Overall, CNA can be considered a safe procedure (71). However, also for CNA the typical complications of invasive procedure are reported, such as pericardial effusion, thrombotic events, vascular complications (AV fistula, pseudoaneurysm); Also induction of Postural Orthostatic Tachycardia Syndrome (POTS) and isolated cases of acute occlusion of the sinus node artery are reported (72), to avoid which reducing the contact force may improve the safety of the procedure. Moreover, targeting the posterior aspect of the SVC could lead to phrenic nerve injury.

However biological long-term effects of CNA are still not well defined. An extensive parasympathetic denervation could determine a durable imbalance in autonomic cardiac regulation, and the sympathetic predominance associated to a higher heart rate may have long-term deleterious effects (73). The loss of sympatho-vagal balance may determine also endothelial (74) and cardiac metabolic dysfunction (71). Furthermore, in a canine model cardiac autonomic denervation determined a reduction of atrial effective refractory period, promoting arrhythmia inducibility (75, 76).

Reinnervation is a theoretical limitation to the efficacy of CNA, because it could be associated to a recovery of the vagal hyperactivity. This is a natural process that physiologically occurs in the first year, considering the massive distribution of nerve endings and the possible overlap of fibers from different GPs, or from the numerous surrounding micro-GP (77). However, a rate of reinnervation up to 30%–50% seems not able to reduce the long-term efficacy of CNA (78). On the other hand, reinnervation may be partially desirable, allowing the repair of the damage on axonal ends of sympathetic and parasympathetic fibers, and avoiding an excessive imbalance in autonomic cardiac regulation (67).

## Other applications of CNA

The CNA may be performed as an alternative to cardiac pacing in patients with atrio-ventricular block (AVB) or sinus bradycardia due to parasympathetic hyperactivity (13, 79, 80).

These functional disorders are often paroxysmal, as symptoms usually occur at rest and during sleep and reduce with the exercise. Otherwise, in case of persistent advanced degree AVB, complete resolution of atrio-ventricular block during atropine administration or exercise test can be considered for differential diagnosis of functional AVB.

Several cases of CNA for treatment of I-II degree functional AV block and sinus bradycardia are reported in literature, especially in young and pediatric patients, avoiding an inappropriate pacemaker implantation (6, 81).

In these patients, considering the previously mentioned specific innervation of SAN and AVN, selective denervation of only the necessary ganglia, respectively RSGP and LIGP, could be a good strategy to make the procedure simpler and more precise. Indeed, selective denervation of RSGP for SAN dysfunction showed satisfactory results at 1 year follow-up (82), and most importantly ageing doesn't seem to affect the acute procedural success of CNA (83).

As for the AV node dysfunction, the selective denervation of PMLGP around the perimitral region of the inferior left atrium seems to reach good results (84). Nevertheless, the AV node may receive innervation from many other ganglionic plexuses, thus a bi-atrial approach seems to be more effective, as demonstrated by the multicentric international registry, PIRECNA study, where 90% of patients received biatrial ablation and 96% of procedural success was reached (80).

### Pediatric population

The CNA seems to be safe also for pediatric patients, as reported by some case reports in the last years, and has been used not only for the treatment of cardioinhibitory syncope, but also for pathological symptomatic bradycardia (85), functional sinus node dysfunction and paroxysmal atrioventricular block (86).

### Gaps in evidence and future perspectives

The CNA may be a valid option for patients aged <40 years with recurrent cardioinhibitory syncope, as a PMK implantation is not recommended in this age group, although there are not yet data from any controlled clinical trials that strongly confirm its effectiveness compared to that of pacing in young individuals.

Eventually, an individualized approach may be dedicated to patients aged between 40 and 60 years. Of note, the age does not impact the outcomes of CNA, if a careful selection of patients is performed (83). Furthermore, CNA could be considered as an alternative to cardiac pacing in case of functional AV block and of recurrent swallowing syncope or carotid sinus syndrome, in absence of intrinsic sinoatrial or AVN dysfunction, in elderly healthy patients aged >60 years (30, 87, 88).

However, the emerging evidence of CNA effectiveness is mainly based on case reports or observational studies. Further investigations and the results of large multicentric prospective registries as the CAN_FWRD registry (45), are highly awaited to evaluate the long-term efficacy and the safety of this procedure.
